# Multiple measurements of the urinary sodium-to-potassium ratio strongly related home hypertension: TMM Cohort Study

**DOI:** 10.1038/s41440-019-0335-2

**Published:** 2019-09-27

**Authors:** Mana Kogure, Takumi Hirata, Naoki Nakaya, Naho Tsuchiya, Tomohiro Nakamura, Akira Narita, Ken Miyagawa, Hiroshi Koshimizu, Taku Obara, Hirohito Metoki, Akira Uruno, Masahiro Kikuya, Junichi Sugawara, Shinichi Kuriyama, Ichiro Tsuji, Shigeo Kure, Atsushi Hozawa

**Affiliations:** 1 Tohoku Medical Megabank Organization, Tohoku University, Sendai, Japan; 2 Tohoku University Graduate School of Medicine, Sendai, Japan; 3 Saitama Prefectural University, Koshigaya, Japan; 4 OMRON Healthcare Co., Ltd, Kyoto, Japan; 5 Tohoku University Hospital, Tohoku University, Sendai, Japan; 6 Tohoku Medical and Pharmaceutical University, Sendai, Japan; 7 Teikyo University School of Medicine, Tokyo, Japan; 8 International Research Institute of Disaster Science, Tohoku University, Sendai, Japan

**Keywords:** Urinary Na/K ratio, Home hypertension, Multiple measurements, TMM Cohort Study

## Abstract

Previous studies have reported a positive association between the urinary sodium-to-potassium (Na/K) ratio and hypertension, and multiple measurements of the casual urinary Na/K ratio are more strongly correlated with the 24-h urinary Na/K ratio than a single measurement. Multiple measurements of the urinary Na/K ratio might be more strongly associated with hypertension. We aimed to determine the association between multiple measurements of the casual urinary Na/K ratio and home hypertension compared with a single measurement. A population-based cross-sectional study was performed in Miyagi Prefecture, Japan. Subjects were over 20 years old and participated in the Tohoku Medical Megabank Project Cohort Study. We targeted 3273 subjects who borrowed home blood pressure (HBP) monitors and urinary Na/K ratio monitors for 10 consecutive days. The association between the urinary Na/K ratio and home hypertension (HBP ≥ 135/85 mmHg or under treatment for hypertension) was examined using multiple logistic regression models. To compare the prediction of home hypertension using multiple measurements with that using a single measurement, we calculated the area under the receiver operating characteristic curve (AUROC). Multiple measurements of the urinary Na/K ratio strongly related to home hypertension were better than 1 or 2 days of measurement (adjusted odds ratio of home hypertension per unit increase in urinary Na/K ratio over 6 days: 1.13–1.15). The AUROC of the urinary Na/K ratio measurement for home hypertension was stable after 5 days (AUROC = 0.779). In conclusion, multiple measurements of the urinary Na/K ratio are strongly related to home hypertension. This finding suggests that multiple measurements of the urinary Na/K ratio are useful for evaluating home hypertension.

## Introduction

The prevention of hypertension is very important because hypertension leads to serious diseases, such as heart disease and stroke [1]. High sodium intake is an established risk factor for hypertension [2–4]. It is also widely reported that potassium intake is inversely associated with hypertension. Recently, the Prospective Urban Rural Epidemiology study showed a positive association between estimated 24-h urinary sodium-to-potassium ratios (Na/K ratios) and both systolic blood pressure (SBP) and diastolic blood pressure (DBP) [5]. The INTERSALT Cooperative Research Group reported that the 24-h urinary Na/K ratio had an even greater effect than sodium or potassium alone on SBP [2]. The 2017 American College of Cardiology/American Heart Association Hypertension Guideline recommends reducing sodium intake and increasing potassium intake to prevent and treat hypertension [6]. Accordingly, the balance between sodium intake and potassium intake (Na/K ratio) has received significant attention in the past year [7].

**Fig. 1:**
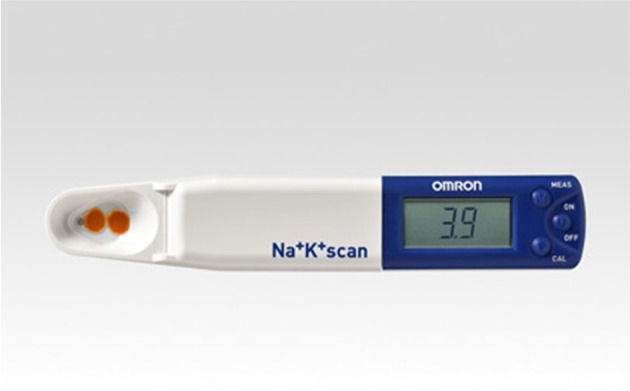
Handy-sized urinary Na/K ratio monitors (HEU-001F; OMRON Healthcare Co., Ltd., Kyoto, Japan)

Recently, OMRON Healthcare Co., Ltd. (Fig. 1, HEU-001F; OMRON Healthcare Co., Ltd., Kyoto, Japan) developed a handy-sized urinary Na/K ratio monitor that can quickly and easily measure the Na/K ratio from urine samples. Thus, this monitor might be useful as a noninvasive, self-monitoring device to estimate the urinary Na/K ratio [8, 9]. However, few studies have shown whether urinary Na/K ratios from self-monitoring devices are associated with blood pressure (BP) or hypertension in the general population.

Self-monitoring devices increase the ease of determining Na/K ratios and can enable multiple readings to be taken, which provides unique benefits. In general, an increasing number of measurement days increases the reproducibility of the urinary Na/K ratio. One study reported that Na/K ratios from casual urine samples might yield more accuracy when repeatedly measured due to the reduction in measurement errors [10]. Another study reported that the urinary Na/K ratio from six random casual urine samples on different days was well correlated with Na/K ratios from seven consecutive 24-h urine samples [11]. A previous study reported that an estimated 24-h urinary Na/K ratio from repeated casual urine samples collected yearly for 5 years was positively associated with blood pressure [12]. Thus, it is feasible that multiple measurements of the urinary Na/K ratio might yield more accurate results. However, no studies have evaluated the relationship between multiple measurements of urinary Na/K ratios and home hypertension in a large population.

Therefore, we aimed to determine whether multiple measurements of the urinary Na/K ratio via casual urine strengthen the relationship between the urinary Na/K ratio and home hypertension better than a single measurement of the casual urinary Na/K ratio.

## Methods

### Participant recruitment

The present study was a cross-sectional study using data from the Tohoku Medical Megabank Project Cohort Study. In 2011, the Tohoku Medical Megabank Project Cohort Study (TMM Cohort Study) aimed to comprehend and address the mental and physical impacts of the Great East Japan Earthquake. In addition, this study sought to improve health and medical care in this region. All subjects were community residents of the Miyagi Prefecture or Iwate Prefecture and were recruited for this study between May 2013 and March 2016 (approval number of the Ethical Review Board: 2016-4-054, HG H25-2).

Those who participated in the baseline survey of the TMM Cohort Study were invited to complete a secondary survey. Starting in June 2017, participants could visit any of the seven Community Support Centers within the Miyagi Prefecture to complete this secondary survey. We also began a collaborative study in June 2017 with OMRON Healthcare Co., Ltd. for participants in the secondary survey of the TMM Cohort Study (June 2017–March 2020). At three of the seven Community Support Centers, we lent urinary Na/K ratio monitors and BP monitors to the participants. Of 7772 participants who agreed to participate in the secondary survey of the TMM Cohort Study at three Community Support Centers before October 2018, 4733 participants agreed to participate in the collaborative study with OMRON Healthcare Co., Ltd. (response rate: 60.9%). All participants provided written, informed consent to participate in this collaborative study as approved by the institutional Review Board of Tohoku Medical Megabank Organization (approval number: 2017-4-007).

### Participation criteria

To be included in the analysis, participants were required to be a previous participant in the TMM Cohort Study and be over 20 years old. Participants with no urinary Na/K ratio data for 10 consecutive days as the maximum measurement period (*n* = 970), no data of home blood pressure (HBP) in the morning (*n* = 10), no information regarding treatment for hypertension (*n* = 465), no listed height or weight (*n* = 5), or no reported alcohol status (*n* = 10) were excluded from the analyses. Overall, a total of 3273 participants fulfilled all criteria and had their data included in the analyses.

### Urinary Na/K ratio data collection

In this survey, participants were provided handy-sized urinary Na/K ratio monitors that were used for 10 consecutive days. These monitors automatically record Na/K ratio measurement values. Participants measured urinary Na/K ratios by themselves in the morning after waking up and in the evening before going to sleep. The staff at each Community Support Center provided face-to-face instructions to each participant on how to use the urinary Na/K ratio monitors; participants were also requested not to lend the monitor to any other person. The monitors were returned to the Community Support Center by mail, and data from the monitors were uploaded to the ToMMo network.

### HBP data collection

In this survey, participants were provided BP monitors (HEM-7080IC; OMRON Healthcare Co., Ltd., Kyoto, Japan) that were used at home for 10 consecutive days during the same period of the urinary Na/K ratio measurement. Participants personally measured HBP in the morning after waking up and in the evening before going to sleep. In the morning, participants were directed to take their HBP within 1 h after waking up, after urination, and before taking drugs and eating breakfast. In the evening, participants measured HBP in a seated position before going to sleep. After 10 days, participants returned the BP monitors to the Community Support Center by mail. The BP monitor data were uploaded to the ToMMo network. Since morning HBP is standard when assessing and measuring hypertension, the average morning HBP for ten days was used for all analyses. The information on treatment for hypertension was obtained by a self-reported questionnaire. The participants chose one option from the following responses: (a) under treatment for hypertension, (b) discontinued hypertension treatment, (c) undertaking lifestyle modification without medication, (d) under observation without medication, or (e) never been diagnosed with hypertension. Individuals who answered (a) were classified as ‘under treatment’, while those who responded with (b)–(e) were classified as ‘without treatment’. Home hypertension was defined as an SBP ≥ 135 mmHg and/or a DBP ≥ 85 mmHg [13] or under treatment for hypertension. As a subanalysis, we also calculated the average of the morning and evening HBP measurements as home hypertension.

### Covariate factors

We included potential confounding factors such as age, sex, body mass index (BMI), and drinking status. Age was determined at the time of consent for the secondary survey. Sex was self-identified using the information from the consent form. BMI was calculated from the height and weight obtained from the self-administered questionnaire. BMI was calculated using the following formula: weight (kg)/height (m^2^).

Drinking status (frequency and amount per day) was determined by the self-administered questionnaire and was classified into four categories: current-drinker, ex-drinker, never-drinker, and cannot drink constitutionally. The type of alcohol was classified into the following six categories: sake, distilled spirits, shochu-based beverages, beer, whiskey, and wine. The frequency of alcohol intake was classified into the following six categories: almost never, 1–3 days/month, 1–2 days/week, 3–4 days/week, 5–6 days/week, or daily. The participants answered how much of each type of alcohol they drank. Each type of alcohol intake was multiplied by the frequency and amount, and converted to the amount of ethanol. The amount of alcohol consumed was classified into the following four categories: <23 g/day, ≥23 g/day, ex-drinker, and never-drinker. We determined the cutoff value of the alcohol amount as 23 g because it is the traditional Japanese unit of sake.

### Calculations and statistical analysis

Another study reported that the urinary Na/K ratio from six random casual urine samples on different days was well correlated with the Na/K ratios from seven consecutive 24-h urine samples [11]. Consequently, we used the average of the morning and evening urinary Na/K ratios, not the urinary Na/K ratio of the morning or evening, in this study. The daily urinary Na/K ratio was calculated as the average of the morning and evening urinary Na/K ratios. If the urinary Na/K ratio was measured only once per day, the value was adopted as the daily value.

In terms of baseline characteristics, we classified participants’ average Na/K ratio on day 10 into quartiles. We tested for trends in urinary Na/K ratios based on patient characteristics, including age, sex, BMI, SBP, DBP, home hypertension, smoking status, and drinking status. We performed trend tests to evaluate the linear relationship between the urinary Na/K ratio and the above variables. We used a general linear model for age, BMI, SBP, and DBP as continuous variables and a logistic regression model for sex, home hypertension, smoking status, and drinking status as categorical variables.

We calculated the mean of all previous days’ daily measurements (up to the stated day) to determine the influence of multiple measurements on the relationship between the urinary Na/K ratio and home hypertension.

To examine the relationship between the urinary Na/K ratio and home hypertension, we applied multiple logistic regression models and calculated the adjusted odds ratio (aOR) with a 95% confidence interval (CI). Adjusted odds ratio *P* values for linear trends were calculated using the quartiles of urinary Na/K ratios. We included potential confounding factors such as age, sex, BMI, and drinking status.

To assess whether multiple measurements of urinary Na/K ratios from casual urine increased the ability to predict home hypertension, we calculated the area under the receiver operating characteristic curve (AUROC) and the 95% CI.

We stratified the participants into two groups: ‘under treatment’ (*n* = 841) and ‘without treatment’ (*n* = 2432) to consider the influence of treatment status on hypertension. We performed multiple logistic regression analysis and calculated the AUROC for each group. In both analyses, we included age, sex, BMI, and drinking status.

All analyses were performed using SAS version 9.4 for Windows (SAS Inc., Cary, NC, USA).

## Results

Overall, a total of 3273 participants fulfilled all criteria and had their data included in the analyses.

Table 1 shows the baseline characteristics of participants grouped by their average 10 days urinary Na/K ratio. Although the difference in BMI between Q1 and Q2, Q4 was not very large, BMI was positively associated with the urinary Na/K ratio (*P* for linear trend <0.001). Men, current smokers, and drinkers (≥23 g/day) were more likely to have an increased urinary Na/K ratio (*P* for linear trend <0.001). However, age was inversely associated with the urinary Na/K ratio (*P* for linear trend <0.001).

**Table 1:** Participants’ baseline characteristics according to urinary Na/K ratio

	Urinary Na/K ratio over 10 days				*P f*or linear trend
	Q1 (<3.27)	Q2 (3.27–4.09)	Q3 (4.10–5.14)	Q4 (≥5.15)	
Number	816	812	822	823	
Age (year) (mean ± SD)	64.5 ± 10.7	63.2 ± 12.2	61.2 ± 13.2	59.3 ± 13.3	<0.001
Sex (number, %)					
Men	210 (25.7)	238 (29.3)	288 (35.0)	313 (38.0)	<0.001
Women	606 (74.3)	574 (70.7)	534 (65.0)	510 (62.0)	<0.001
BMI (mean ± SD)	22.5 ± 3.1	23.0 ± 3.3	23.3 ± 3.3	23.5 ± 3.5	<0.001
Urinary Na/K ratio (median, IQR)	2.8 (2.4–3.0)	3.7 (3.5–3.9)	4.6 (4.3–4.8)	6.0 (5.5–6.8)	–
SBP (mmHg) (mean ± SD)	124.5 ± 15.9	126.2 ± 15.8	126.9 ± 16.4	129.4 ± 17.0	<0.001
DBP (mmHg) (mean ± SD)	73.6 ± 9.5	74.2 ± 9.1	74.8 ± 9.8	77.6 ± 9.9	<0.001
Home hypertension^a^ (number, %)	326 (40.0)	369 (45.4)	368 (44.8)	407 (49.5)	<0.001
Smoking status (number, %)					
Current-smoker	36 (4.4)	40 (4.9)	65 (7.9)	91 (11.1)	<0.001
Ex-smoker	169 (20.7)	225 (27.7)	236 (28.7)	256 (31.1)	<0.001
Non-smoker	607 (74.4)	541 (66.6)	517 (62.9)	474 (57.6)	<0.001
Unknown	4 (0.5)	6 (0.7)	4 (0.5)	2 (0.2)	–
Drinking status (number, %)					
<23 g/day	220 (27.0)	238 (29.3)	250 (30.4)	242 (29.4)	0.235
≥23 g/day	99 (12.1)	136 (16.8)	166 (20.2)	225 (27.3)	<0.001
Ex-drinker	25 (3.1)	24 (3.0)	12 (1.5)	12 (1.5)	0.007
Never-drinker	472 (57.8)	414 (51.0)	394 (47.9)	344 (41.8)	<0.001

*BMI* body mass index, *Na/K* sodium potassium ratio, *IQR* interquartile range, *SBP* systolic blood pressure, *DBP* diastolic blood pressure

^a^Home hypertension was defined as an SBP ≥ 135 mmHg and/or a DBP ≥ 85 mmHg or under treatment for hypertension

**Fig. 2:**
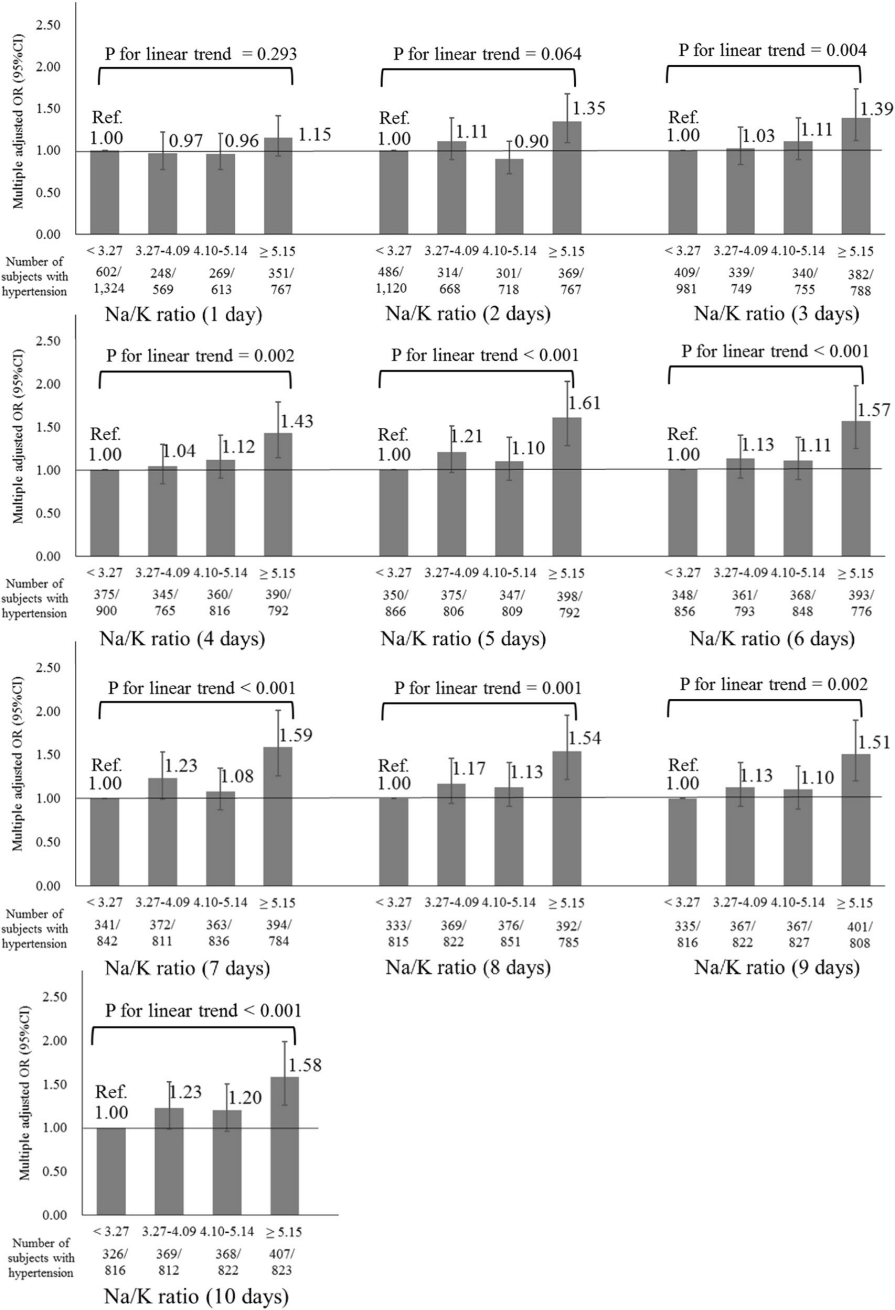
Relationship between 1 and 10 days average urinary Na/K ratios and home hypertension. *P* values for linear trends were derived from multiple logistic regression analysis. Error bars represent 95% confidence intervals

Figure 2 shows the relationship between the average urinary Na/K ratio and home hypertension from 1 to 10 days. The urinary Na/K ratio was not associated with home hypertension on days 1 or 2 (*P* for linear trend on 1 day = 0.293, *P* for linear trend on 2 days = 0.064). However, multiple measurements increased the robustness of the relationship. Although we also calculated the average of morning and evening HBP as home hypertension, our result was essentially unchanged (data not shown).

**Fig. 3:**
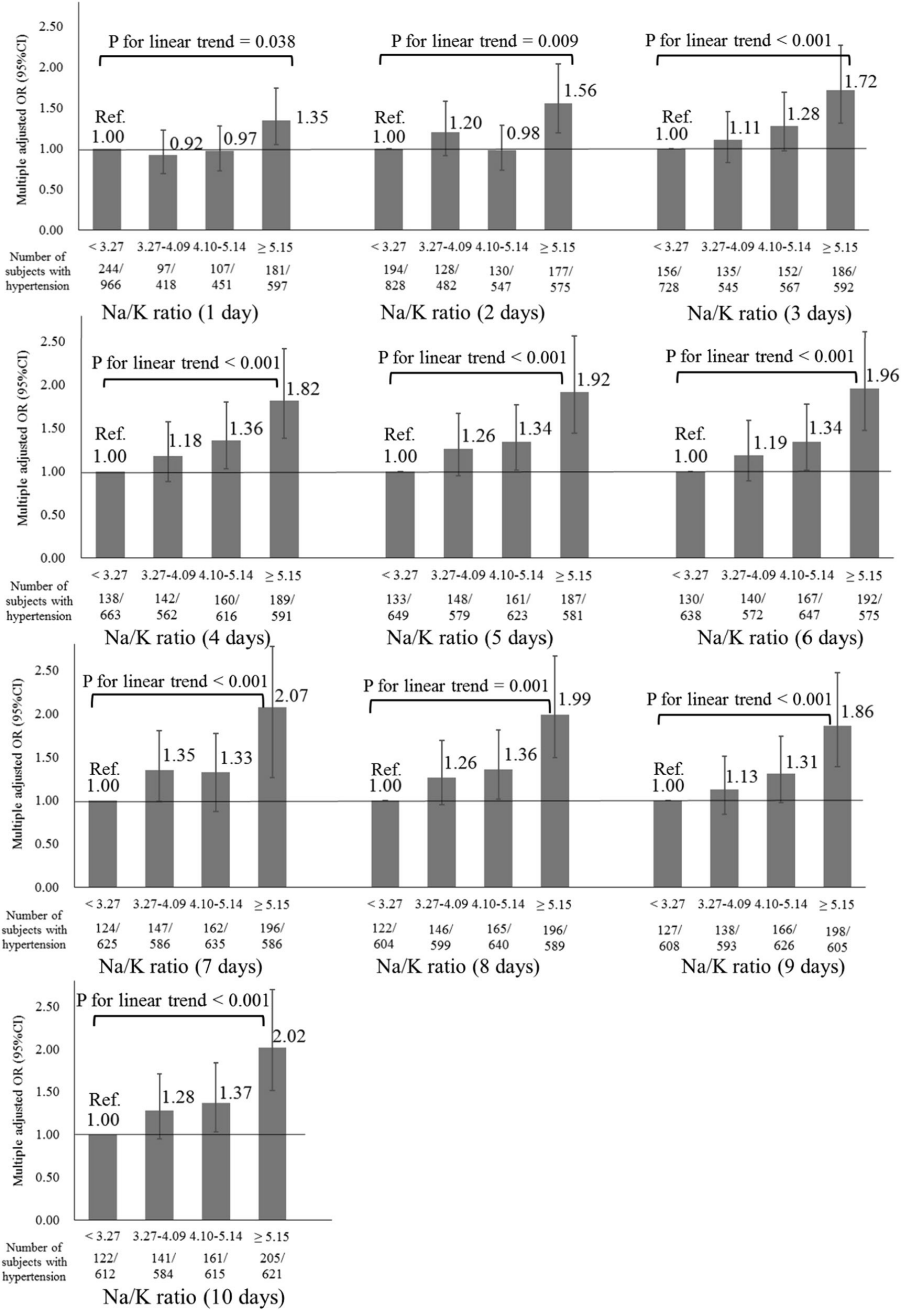
Relationship between 1 day and 10 days average urinary Na/K ratios and home hypertension according to without treatment for hypertension. *P* values for linear trends were derived from multiple logistic regression analysis. Error bars represent 95% confidence intervals

Figure 3 shows the relationship between the average urinary Na/K ratio and home hypertension from 1 to 10 days according to without treatment for hypertension. The urinary Na/K ratio was positively associated with home hypertension on 1 day in the without treatment group (*P* for linear trend = 0.038). Multiple measurements increased the robustness of the relationship regardless of hypertension treatment status. The average 10 days urinary Na/K ratio was associated with a higher SBP (regression coefficient of average 10 days urinary Na/K ratio = 1.47, *p* < 0.001). This finding was independent of age, sex, BMI, and alcohol intake (regression coefficients for age, sex (ref. women), BMI, and alcohol intake were 0.44, −3.85, 1.39, and 0.06, respectively; all variables were *p* < 0.001).

**Fig. 4:**
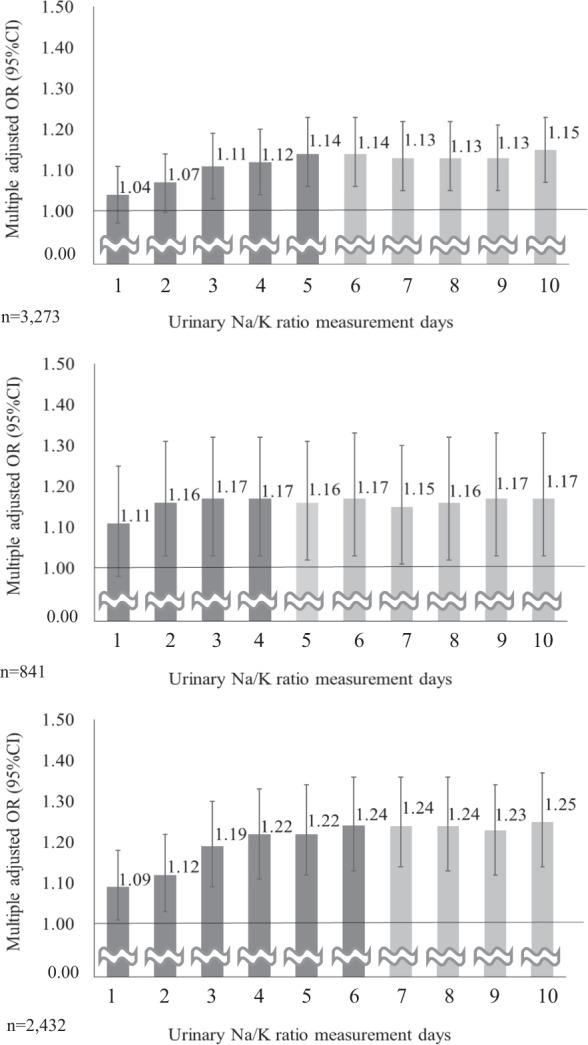
The prevalence of home hypertension per unit increase in average urinary Na/K ratios from 1 to 10 days (above: overall, middle: under treatment for hypertension, below: without treatment for hypertension). Error bars represent 95% confidence intervals

Figure 4 shows the prevalence of home hypertension per unit increase in average urinary Na/K ratios from 1 to 10 days. Overall, the aOR increased from 1 to 5 days; however, the aOR was stable after 6 days (range of 1.13–1.15). In the without treatment group and the under treatment for hypertension group, the aOR increased until 4–6 days. However, the aOR was stable after 5–7 days.

Table 2 shows the AUROC for multiple measurements of the urinary Na/K ratio to predict home hypertension. Overall, the AUROC of the urinary Na/K ratio measurement for home hypertension increased until reaching 3–5 days’ worth of measurements. However, the AUROC was stable after 5 days (AUROC = 0.779). In the under treatment group, the AUROC of the urinary Na/K ratio measurements for home hypertension fluctuated between 0.600 and 0.606 after 3 days. In the without treatment group, the AUROC of the urinary Na/K ratio measurements for home hypertension increased until 7 days of measurement. However, the AUROC was stable after 8 days (AUROC = 0.742–0.743).

**Table 2:** Area under the ROC curve for multiple measurements of urinary Na/K ratios to predict home hypertension

	Area under the ROC curve (95% CI)^a^		
	Overall	Under treatment	Without treatment
	(*n* = 3,273)	(*n* = 841)	(*n* = 2432)
**Measurement day**			
**1**	0.777 (0.762–0.793)	0.597 (0.558–0.635)	0.739 (0.717–0.761)
**2**	0.778 (0.763–0.794)	0.599 (0.561–0.637)	0.740 (0.718–0.761)
**3**	0.778 (0.762–0.794)	0.604 (0.565–0.642)	0.741 (0.719–0.762)
**4**	0.778 (0.763–0.794)	0.600 (0.562–0.639)	0.741 (0.720–0.763)
**5**	0.779 (0.763–0.794)	0.603 (0.565–0.642)	0.741 (0.720–0.763)
**6**	0.779 (0.763–0.794)	0.606 (0.567–0.644)	0.742 (0.721–0.764)
**7**	0.779 (0.763–0.795)	0.603 (0.565–0.642)	0.743 (0.722–0.765)
**8**	0.779 (0.763–0.795)	0.600 (0.562–0.639)	0.743 (0.721–0.764)
**9**	0.779 (0.763–0.794)	0.603 (0.565–0.641)	0.742 (0.720–0.763)
**10**	0.779 (0.764–0.795)	0.604 (0.566–0.643)	0.743 (0.722–0.765)

*Na/K* sodium potassium ratio, *CI* confidence interval, *ROC* receiver operating characteristic curve

^a^Adjusted for age (continuous variable), sex, BMI (continuous variable), and drinking status (<23 g/day, ≥23 g/day, ex-drinker, never-drinker)

## Discussion

We investigated whether multiple measurements of the urinary Na/K ratio in casual urine strengthened the relationship between the urinary Na/K ratio and home hypertension to a greater extent than a single measurement of the casual urinary Na/K ratio during the same period. The urinary Na/K ratio measured using the self-monitoring device was not associated with increased home hypertension based on the 1 or 2 days of measurements. However, the urinary Na/K ratio was positively associated with home hypertension after 3 days of measurement. In addition, the AUROC of the urinary Na/K ratio measurement for home hypertension increased until 5 days of measurement. Furthermore, the AUROC of the urinary Na/K ratio measurement for hypertension was stable after 5 days.

Previous reports have shown a positive association between 24-h urinary Na/K ratios and BP or hypertension [2, 14–16]. Another study reported that an estimated 24-h urinary Na/K ratio from repeated casual urine samples collected yearly within 5 years was positively associated with blood pressure [12]. However, this study is the first to show that the mean urinary Na/K ratio measured during 10 consecutive days by a self-monitoring device was associated with home hypertension in the general population.

A previous study compared the urinary Na/K ratio of six random casual urine catches on different days to the average urinary Na/K ratio from seven consecutive days of 24-h urine samples. The correlation coefficients of each consecutive day between the average urinary Na/K ratio of random casual urine for up to 7 days and the average urinary Na/K ratio for 7 consecutive days of 24-h urine were 0.51, 0.64, 0.71, 0.82, 0.85, 0.87, and 0.87, respectively [11], i.e., the correlation coefficient increased up to 5 days and then remained stable at approximately 0.87. Another study reported that multiple measurements of urine samples have been required because 24-h urine sample which was a single urine sample could not predict sodium and potassium [17, 18]. Our results were consistent with those of this previous study. Together, these findings support the importance of using multiple measurements for the urinary Na/K ratio when using self-monitoring devices. Specifically, our results recommend taking measurements on multiple days (more than 5–6 days if possible) to obtain optimal accuracy.

In the without treatment group, the relationship between the average urinary Na/K ratio from 1 to 10 days of measurement and home hypertension was more apparent than the relationship in the under treatment group. The participants under treatment may receive instruction regarding lifestyle modification from health care staff, such as a doctor or public nurse. Participants with high BP in particular might have been recommended to reduce their sodium intake and increase their potassium intake (lower Na/K ratio). However, even in the under treatment group, the urinary Na/K ratio was positively associated with home hypertension after 2 days (four individual measurements). Thus, we considered that multiple measurements of the urinary Na/K ratio are useful for evaluating home hypertension, even for individuals who are under treatment. On the other hand, the aOR for home hypertension in the highest quartile of the urinary Na/K ratio was approximately twice as high as the aOR for home hypertension in the lowest quartile of the urinary Na/K ratio after 5 days (10 individual measurements) in the without treatment group. Therefore, we considered that a high urinary Na/K ratio is a very strong risk factor for hypertension among participants who are not using antihypertensive medication.

The present study had several strengths. First, this is the first report to explore the relationship between multiple measurements of urinary Na/K ratios obtained through a self-monitoring device and home hypertension. Previous studies have reported that HBP strongly predicts the risk of stroke when compared to casually measured BP [19]. This better prediction might be due to the lack of the white coat effect or the ability to obtain multiple measurements. In addition, the compliance of participants using urinary Na/K ratio monitors was high. Of the 4733 men and women who agreed to participate in this study, 3763 (79.5%) measured the Na/K ratio for all 10 days. However, participants who measured their urinary Na/K ratio every day might be inherently more conscientious of their lifestyle than the general population. In fact, in our study, participants who took measurements for the full 10 days had a lower mean Na/K ratio (4.38) than the mean Na/K ratio of participants who had to be excluded from the study due to incomplete data (4.85). Finally, our study included various confounding factors.

Our study has some limitations. First, it is difficult to confirm causality since this study had a cross-sectional design. Considering that participants with high BP would be unlikely to increase their Na consumption, we considered that lifestyle modifications for high BP would lead to an underestimated relationship between a high Na/K ratio and home hypertension. Second, we could not assess antihypertensive drug status since we have not yet digitized all drug information. However, the treatment status for hypertension was assessed. If we could have evaluated the intake of specific antihypertensive drugs, our findings regarding the effect of the urinary Na/K ratio on home hypertension would likely have been more robust. In the future, we will include the hypertension treatment status in this study. Finally, we did not directly assess whether the predictive power of the long-term casual urinary Na/K ratio might be equivalent to that of a 1 day 24-h urinary Na/K ratio. However, a previous study reported that the casual urinary Na/K ratio of six random days was strongly correlated with the average urinary Na/K ratio from seven consecutive days of 24-h urine samples [11]. Thus, we believe that our findings are applicable.

In conclusion, multiple measurements of the urinary Na/K ratio were strongly related to home hypertension regardless of the treatment status for hypertension. We suggest that multiple measurements of the urinary Na/K ratio are desirable for assessing home hypertension because long-term measurements of the urinary Na/K ratio might reflect participants’ dietary balance of the Na/K ratio. We believe that noninvasive self-monitoring devices for the urinary Na/K ratio are effective tools to estimate the risk of hypertension and to evaluate the effect of salt-intake restriction in clinical settings or in the public health field. Thus, we expect that self-monitoring devices will facilitate the management and prevention of hypertension in the future.

## References

[1] Forouzanfar MH, Liu P, Roth GA, Ng M, Biryukov S, Marczak L (2017). Global burden of hypertension and systolic blood pressure of at least 110 to 115 mm Hg, 1990–2015. JAMA..

[2] Intersalt Cooperative Research Group. Intersalt: an international study of electrolyte excretion and blood pressure. Results for 24 hour urinary sodium and potassium excretion. BMJ. 1988;297:319–28. 10.1136/bmj.297.6644.319 PMC1834069 3416162

[3] Aburto NJ, Ziolkovska A, Hooper L, Elliott P, Cappuccio FP, Meerpohl JJ (2013). Effect of lower sodium intake on health: systematic review and meta-analyses. BMJ..

[4] He FJ, Li J, Macgregor GA (2013). Effect of longer term modest salt reduction on blood pressure: Cochrane systematic review and meta-analysis of randomised trials. BMJ..

[5] Mente A, O’Donnell MJ, Rangarajan S, McQueen MJ, Poirier P, Wielgosz A (2014). Association of urinary sodium and potassium excretion with blood pressure. N Engl J Med.

[6] Whelton PK, Carey RM, Aronow WS, Casey DE, Collins KJ, Dennison Himmelfarb C (2018). 2017 ACC/AHA/AAPA/ABC/ACPM/AGS/APhA/ASH/ASPC/NMA/PCNA guideline for the prevention, detection, evaluation, and management of high blood pressure in adults: executive summary: a report of the american college of cardiology/american heart association task force on clinical practice guidelines. J Am Soc Hypertens..

[7] Iwahori T, Miura K, Ueshima H (2017). Time to consider use of the sodium-to-potassium ratio for practical sodium reduction and potassium increase. Nutrients..

[8] Iwahori T, Ueshima H, Ohgami N, Yamashita H, Miyagawa N, Kondo K (2018). Effectiveness of a self-monitoring device for urinary sodium-to-potassium ratio on dietary improvement in free-living adults: a randomized controlled trial. J Epidemiol..

[9] Yatabe MS, Iwahori T, Watanabe A, Takano K, Sanada H, Watanabe T (2017). Urinary sodium-to-potassium ratio tracks the changes in salt intake during an experimental feeding study using standardized low-salt and high-salt meals among healthy japanese volunteers. Nutrients..

[10] Iwahori T, Miura K, Ueshima H, Chan Q, Dyer AR, Elliott P (2017). Estimating 24-h urinary sodium/potassium ratio from casual (‘spot’) urinary sodium/potassium ratio: the INTERSALT study. Int J Epidemiol.

[11] Iwahori T, Ueshima H, Miyagawa N, Ohgami N, Yamashita H, Ohkubo T (2014). Six random specimens of daytime casual urine on different days are sufficient to estimate daily sodium/potassium ratio in comparison to 7-day 24-h urine collections. Hypertens Res.

[12] Thi Minh Nguyen T, Miura K, Tanaka-Mizuno S, Tanaka T, Nakamura Y, Fujiyoshi A (2019). Association of blood pressure with estimates of 24-h urinary sodium and potassium excretion from repeated single-spot urine samples. Hypertens Res..

[13] Shimamoto K, Ando K, Fujita T, Hasebe N, Higaki J, Horiuchi M (2014). The Japanese Society of hypertension guidelines for the management of hypertension (JSH 2014). Hypertens Res..

[14] Stamler J, Rose G, Stamler R, Elliott P, Dyer A, Marmot M (1989). INTERSALT study findings. Public health and medical care implications. Hypertension..

[15] Cook NR, Kumanyika SK, Cutler JA (1998). Effect of change in sodium excretion on change in blood pressure corrected for measurement error. The Trials of Hypertension Prevention, Phase I. Am J Epidemiol..

[16] Binia A, Jaeger J, Hu Y, Singh A, Zimmermann D (2015). Daily potassium intake and sodium-to-potassium ratio in the reduction of blood pressure: a meta-analysis of randomized controlled trials. J Hypertens..

[17] Birukov A, Rakova N, Lerchl K, Olde Engberink RH, Johannes B, Wabel P (2016). Ultra-long-term human salt balance studies reveal interrelations between sodium, potassium, and chloride intake and excretion. Am J Clin Nutr..

[18] Lerchl K, Rakova N, Dahlmann A, Rauh M, Goller U, Basner M (2015). Agreement between 24-hour salt ingestion and sodium excretion in a controlled environment. Hypertension..

[19] Ohkubo T, Asayama K, Kikuya M, Metoki H, Hoshi H, Hashimoto J (2004). How many times should blood pressure be measured at home for better prediction of stroke risk? Ten-year follow-up results from the Ohasama study. J Hypertens..

